# Severe cerebral injury secondary to an air embolism following ERCP for biliary calculus extraction: a case report

**DOI:** 10.3389/fmed.2026.1787974

**Published:** 2026-06-26

**Authors:** Weimei Zhou, Liyang Hang, Guodong Wu, Chen Zhang, Xiaoyan Wang, Jiang Liu, Xuhua Cai

**Affiliations:** 1 Department of Gastroenterology, Huzhou Central Hospital, Affiliated Central Hospital of Huzhou University, Huzhou, China; 2 Department of General Medicine, Community Health Service Center of Renhuangshan, Binhu, Huzhou, China

**Keywords:** air embolism, anesthesiology, critical care medicine, endoscopy, ERCP

## Abstract

**Objective:**

This report describes a rare case of severe cerebral injury secondary to an air embolism following endoscopic retrograde cholangiopancreatography (ERCP) with stone extraction. Through meticulous analysis of this complication, we aim to elucidate the potential mechanisms responsible for inadvertent gas entry during therapeutic procedures, evaluate strategies for early detection, and discuss precautionary measures to enhance procedural safety in biliary interventions. This study provides valuable insights into understanding and potentially reducing such adverse iatrogenic outcomes in interventional endoscopy practice.

**Methods:**

This is a retrospective case study describing the full inpatient journey of one patient in the Department of Gastroenterology.

**Results:**

During the ERCP procedure, the patient experienced a decrease in oxygenation and a significant increase in blood pressure. The procedure was temporarily stopped to allow for stabilization of the patient’s condition. Following recovery, the surgical procedure was resumed. However, the patient again experienced decreased oxygenation, accompanied by hypotension and bradycardia. Immediate resuscitation measures were initiated, followed by admission to the critical care medicine (CCM) unit. Despite these interventions, the patient developed severe cerebral injury. Ultimately, following the family’s decision to withdraw treatment, the patient was discharged from the hospital.

## Introduction

ERCP is an indispensable diagnostic and therapeutic modality in the management of biliary diseases and pancreatic diseases. By employing endoscopic techniques, ERCP allows direct visualization of the biliary and pancreatic ducts, thereby enabling accurate diagnosis and effective treatment of various disorders affecting these systems. Its applications are wide ranging and include but are not limited to the removal of biliary stones, dilation of biliary strictures, management of pancreatic duct stones, diagnosis and treatment of biliary tumors, and drainage for pancreatitis. However, it is crucial to acknowledge the potential risks associated with ERCP. One such risk is an air embolism, a severe complication that can cause significant cardiopulmonary dysfunction, cerebral embolism, thromboinflammation, and potentially fatal outcomes (1, 2). We present a case report of a patient who experienced an air embolism during an ERCP stone extraction procedure, leading to severe cerebral injury.

## Case

A 70-year-old female patient with a history of cholecystectomy and choledochoduodenostomy was admitted to our institution because of a 5-day history of fever and 1-day history of abdominal pain. Upon physical examination, the abdomen was soft with tenderness in the epigastric and right upper quadrant regions; however, there was no rebound tenderness or abdominal muscle rigidity. The liver and spleen were not palpable, and mild icterus was noted in the sclera and skin. Laboratory tests revealed elevated bilirubin levels: total bilirubin concentration of 54.5 μmol/L (direct bilirubin concentration of 32.5 μmol/L and indirect bilirubin concentration of 22.0 μmol/L). Contrast-enhanced abdominal CT revealed multiple calculi within the left intrahepatic bile ducts. The patient was ultimately diagnosed with the following conditions:

Intrahepatic bile duct stones complicated by acute cholangitis.
Hepatic insufficiency.
Postcholecystectomy status.
Choledochoduodenostomy.

After a comprehensive anesthetic risk assessment was performed and informed consent was obtained from both the patient and her accompanying family members, it was decided to proceed with endoscopic lithotripsy and stone extraction under ERCP. Preoperative echocardiography showed mild regurgitation of the mitral and tricuspid valves and suboptimal left ventricular diastolic performance (ejection fraction (EF) 70%).

Upon entering the operating suite, the patient was placed in the left lateral decubitus position. Total intravenous anesthesia with preserved spontaneous respiration was administered, supplemented by placement of a nasopharyngeal airway. Supplemental oxygen at 3 L/min was delivered via nasal cannula, and continuous monitoring was instituted, encompassing non-invasive blood pressure, arterial oxygen saturation (SaO₂), and electrocardiography.

Compressed ambient air insufflation was employed to achieve bowel distension during ERCP. The endoscope was smoothly advanced through the esophagus and gastric cavity without any technical difficulty. No esophageal varices were detected in the distal esophagus, and the gastric mucosa appeared generally smooth. A bilioenteric anastomosis with a patent lumen and intact mucosa was identified on the anterior wall of the duodenal bulb near the superior angulation. The endoscope was then advanced across the anastomotic site to perform subsequent interventions. A large impacted calculus measuring approximately 1.5 cm in diameter was found at the acute angulation of the left hepatic duct, with spontaneous drainage of purulent fluid from the ductal lumen (Figure 1). A stone basket and snare were subsequently used to fragment the impacted calculus, and partial stone fragments were extracted.

**Figure 1:**
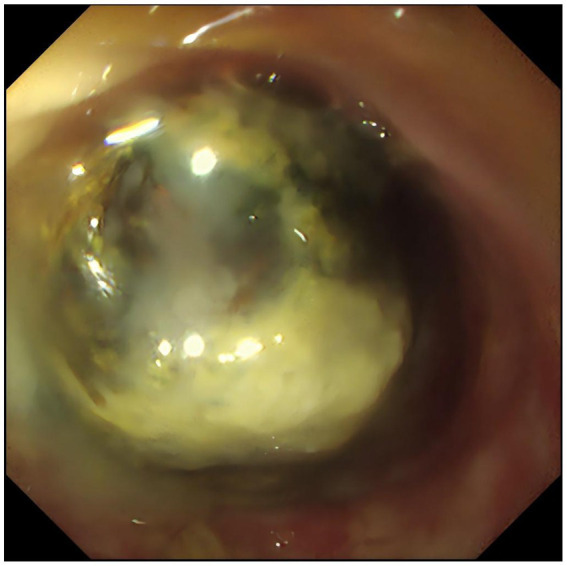
Endoscopic image of the biliary tree showing a calculus within the intrahepatic duct with purulent exudate.

The patient initially maintained hemodynamic stability, with a heart rate of 70–80 beats/min and a mean arterial pressure of 80–90 mmHg.

Approximately 80 min after the initiation of the ERCP procedure, the patient developed acute hypoxemia (SpO₂ nadir: 85%), accompanied by a paroxysmal elevation in blood pressure (systolic peaking at ~200 mmHg). The procedure was immediately suspended, and a bolus dose of 10 mg urapidil was administered intravenously, which resulted in prompt normalization of blood pressure and gradual improvement of oxygenation (SpO₂ 97%). Following fluoroscopic exclusion of pneumothorax and given that the procedure was near completion, a joint decision was made by the surgical and anesthesia teams to resume the intervention.

However, 90 min into the procedure, the patient again experienced a precipitous decline in oxygen saturation. The procedure was halted, and manual bag-valve-mask ventilation was initiated. Despite these measures, oxygenation continued to deteriorate, reaching a nadir of 60%. This prompted urgent repositioning of the patient to the supine position for emergent endotracheal intubation, which was performed following administration of a neuromuscular blocking agent (rocuronium 0.6 mg/kg) per standard protocol.

Concurrently, the patient developed severe bradycardia (heart rate: 37 beats per minute) accompanied by unmeasurable blood pressure, necessitating the immediate initiation of cardiopulmonary resuscitation (CPR) in accordance with Advanced Cardiac Life Support (ACLS) guidelines. Intravenous adrenaline (1 mg, repeated every 3–5 min per ERC guidelines) was administered to augment heart rate and restore hemodynamic stability, supplemented by targeted correction of acidosis with intravenous sodium bicarbonate (50 mmol). During sustained CPR, electrocardiographic monitoring revealed ventricular fibrillation (VF). Emergency electrical defibrillation was promptly performed with an initial energy dose of 200 Joules, followed by immediate resumption of CPR. Defibrillation was repeated at 360 Joules as needed until return of spontaneous circulation (ROSC) was achieved.

After 70 min of sustained resuscitative efforts, sinus rhythm was successfully restored. Initial post-ROSC vital signs were documented as follows: heart rate 108 beats per minute, blood pressure 82/43 mmHg, SpO₂ 68%. Notably, spontaneous respiration did not resume following ROSC, necessitating continued mechanical ventilation via the existing endotracheal tube.

Twenty-five minutes after initial ROSC, despite the restoration of spontaneous cardiac and respiratory function (heart rate 96 bpm, blood pressure 114/68 mmHg, SpO₂ 91% on FiO₂ 55%), the patient developed anisocoria and deep coma. This was accompanied by persistent hemodynamic instability characterized by hypotension refractory to fluid resuscitation, necessitating the initiation of high-dose vasopressor support with intravenous norepinephrine at a rate of 0.3–1.0 μg/kg/min, titrated to maintain a mean arterial pressure ≥ 65 mmHg per ERC guidelines. The following examinations were performed at this time. Suspecting a stroke, urgent brain computed tomography (CT) was performed. CT imaging revealed multifocal pneumocephalus within the intracranial cavity and a vascular air embolism involving the left occipital region (Figure 2). Bedside echocardiography revealed diffuse hypokinesis of the left ventricular wall and impaired left ventricular systolic function (EF46%).

**Figure 2:**
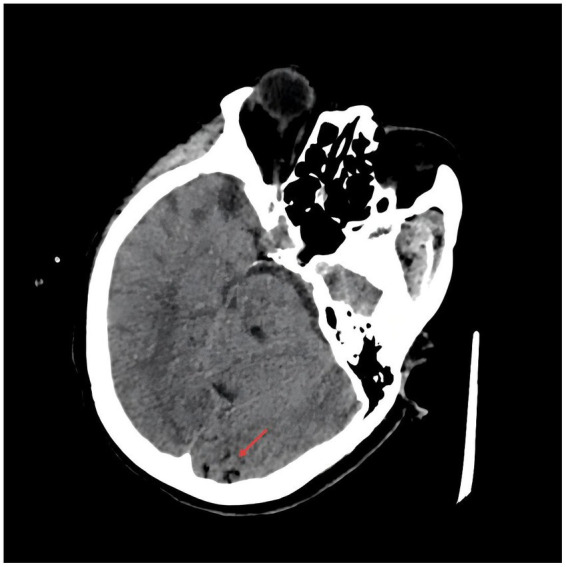
Axial non-contrast brain computed tomography (CT) scan: a hypodense focus in the left cerebral hemisphere is indicated by a red arrow, representing in the site of air embolism.

Immediately following initial resuscitation, the patient was transferred to the Department of Critical Care Medicine (CCM) for further management.

Upon CCM admission, the patient presented with a persistent comatose state. On neurological examination, the pupils were noted to be bilaterally 1.5 mm in diameter and sluggishly reactive to light. Vital signs were as follows: body temperature was maintained at 34.0 °C using an ice pillow as part of targeted temperature management (TTM); heart rate was 83 bpm; blood pressure was 113/62 mmHg under continuous intravenous norepinephrine infusion (0.6 μg/kg/min); and respiratory rate was 16 breaths/min with mechanical ventilation in synchronized intermittent mandatory ventilation (SIMV) mode. Peripheral oxygen saturation (SpO₂) was 99% with a fraction of inspired oxygen (FiO₂) of 55%. For sedation and neuroprotection, the patient was started on a continuous intravenous infusion of propofol at 600 mg/day. Physical examination was notable for abdominal distension and coarse breath sounds with occasional dry rales on pulmonary auscultation. No lower extremity edema was observed. Laboratory tests on admission revealed a normal hemoglobin level (102 g/L) but a markedly elevated white blood cell count (41.9 × 10^9^/L) and increased inflammatory markers (high-sensitivity C-reactive protein, 84.1 mg/L). Platelet count was within the normal range (278 × 10^9^/L). Liver function tests showed markedly elevated transaminases (aspartate aminotransferase, 697.9 U/L; alanine aminotransferase, 224.4 U/L) and hypoalbuminemia (23.8 g/L). Renal function was mildly impaired, with a serum creatinine level of 84.6 μmol/L. Serum electrolytes were within normal limits (potassium, 3.81 mmol/L). Arterial blood gas (ABG) analysis performed on 60% FiO₂ demonstrated a mixed acid–base disorder with a primary metabolic acidosis (HCO₃^−^ 18.9 mmol/L, base excess −4.1 mmol/L) and a superimposed primary respiratory alkalosis (PaCO₂ 24.8 mmHg), resulting in alkalemia (pH 7.490). The PaO₂/FiO₂ ratio was 213, confirming significant hypoxemia.

During the intensive care unit (ICU) stay, the patient received comprehensive supportive care and targeted interventions. Neuroprotective strategies were implemented, including TTM at 34.0 °C to reduce cerebral metabolic rate and mitigate hypoxic–ischemic injury. Propofol sedation was continued for 2 days post-admission but was subsequently discontinued due to worsening of the patient’s neurological status and overall clinical condition. Hemodynamic stability was maintained with a continuous norepinephrine infusion, titrated to ensure adequate cerebral and systemic perfusion pressures. Mechanical ventilation was continued in SIMV mode, with FiO₂ adjusted (40–60%) to maintain SpO₂ ≥ 95%. Supportive care included fluid resuscitation, correction of anemia and hypoalbuminemia, and strict electrolyte balance monitoring. Neurological status was assessed regularly using standardized coma scales.

One week post-surgery, despite maximal supportive therapy, the patient showed no neurological recovery, remaining in a deep coma with bilaterally fixed and dilated pupils-unequivocal signs of a devastating brainstem injury. Cranial CT scans revealed progressive multifocal cerebral infarction, worsening cerebral edema, and ultimately, cerebral herniation, indicative of irreversible global cerebral injury (Figure 3). In view of the extremely poor prognosis, a multidisciplinary team discussion was held with the patient’s family. After thorough deliberation regarding the clinical situation and treatment options, a joint decision was made to withdraw life-sustaining treatments. In accordance with the family’s wishes, the patient was extubated and discharged home under palliative care, with a focus on comfort and dignity.

**Figure 3:**
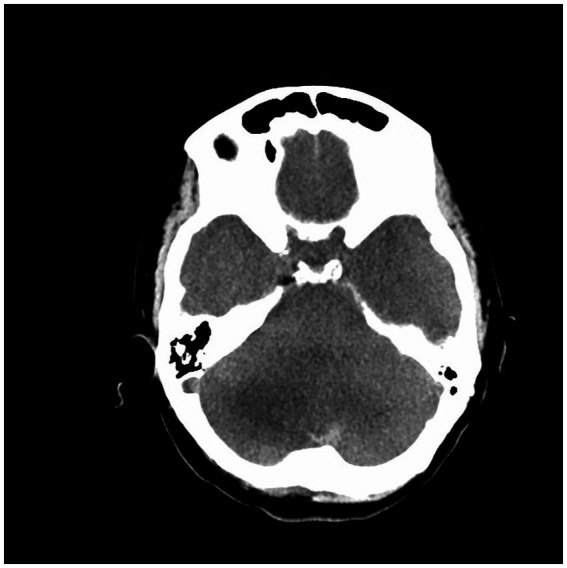
Axial non-contrast computed tomography (CT) image of the brain, showing diffuse cerebral swelling with loss of normal gray–white matter differentiation and effacement of cortical sulci.

## Discussion

ERCP has been extensively utilized in the diagnosis and management of various hepatobiliary and pancreatic diseases. However, the associated complications of ERCP warrant serious consideration. Complications such as post-ERCP pancreatitis, perforation, infection, and hemorrhage have been well documented in the literature (3). In contrast, an air embolism following ERCP remains underreported. This is a rare but potentially life-threatening complication with significant clinical implications (4, 5).

A retrospective study revealed that the incidence of an air embolism following ERCP is approximately 3.32 cases per 100,000 procedures, with a mortality rate as high as 15.4% (6). Survivors may also experience permanent sequelae (6). These findings highlight the severity and potential lethality of an air embolism as a complication, underscoring the critical need for heightened vigilance and preventive measures.

Some scholars posit that endoscopic sphincterotomy (EST) constitutes a significant risk factor for air embolism associated with ERCP, with the majority of previously reported cases of air embolism occurring during this procedural step (7–9). The proposed mechanism involves inadvertent injury or transection of duodenal venous tributaries during sphincterotomy, permitting insufflated gas to dissect along tissue planes within the intestinal wall and subsequently enter the portal venous system.

However, in the present case, EST was not performed; instead, the procedure was completed via a choledochoduodenal anastomosis, representing a distinctive deviation from conventional ERCP technique. Consequently, the occurrence of air embolism in this patient cannot be attributed to gas entry through transected vessels during sphincterotomy. For this patient, the pathogenesis of air embolism may be attributed to the choledochoduodenal anastomosis.

The pre-existing choledochoduodenal anastomosis may have constituted a direct channel for air entry. During ERCP, if intraluminal pressure in the duodenum becomes excessively elevated due to insufflation, a large volume of air may be forced retrogradely through this anastomosis into the biliary tract, subsequently entering the systemic circulation via the hepatic venous system and leading to embolism (10). Moreover, cases of gas embolism caused by air entry through choledochoduodenal fistulas have been reported in the literature (8). These findings indicate that any form of abnormal communication between the biliary and digestive tracts—whether iatrogenic anastomosis or pathological fistula—may compromise the integrity of the biliary system, thereby representing a high-risk anatomical condition predisposing individuals to air embolism.

After entering the bloodstream via the possible pathway described above, the gas should theoretically return with venous blood to the lungs and be filtered out. However, in this patient, this pathway was not followed—the gas ultimately reached the brain, resulting in severe brain injury and death. A possible explanation for this phenomenon is that when the rate or volume of gas injected into the body exceeds a certain threshold, it can breach the filtration barrier of the pulmonary circulation, enter the arterial system, and cause embolism. This mechanism has been widely accepted by many scholars and has been validated in animal experiments (11–14). Given that the presence of the anastomosis in this patient enabled a substantial volume of air to rapidly enter the systemic circulation, this mechanism likely played a significant role in the pathogenesis of the present case.

Another possible mechanism is the presence of a patent foramen ovale (PFO) in the patient. This is a common structural abnormality of the heart through which gas can enter the arterial circulation, leading to paradoxical embolism. Of note, although the foramen ovale may be functionally closed by increased left atrial pressure postnatally in some individuals, anatomical patency persists. Under normal circumstances, because left atrial pressure exceeds right atrial pressure, the patent foramen ovale remains functionally closed without significant shunting, so most patients are asymptomatic and difficult to diagnose. However, when pulmonary circulation pressure rises, right atrial pressure may exceed left atrial pressure, prompting the foramen ovale to open and allowing gas to enter the arterial system, thereby causing cerebral embolism (15). During the occurrence of air embolism, the pressure in the right heart system increases significantly (16), providing the hemodynamic conditions necessary for this mechanism to take effect. Furthermore, such a patent foramen ovale is often difficult to detect using standard transthoracic echocardiography. Therefore, even if bedside ultrasound in this patient did not reveal an obvious patent foramen ovale, this diagnosis cannot be entirely ruled out.

Furthermore, autopsy findings and sporadic case reports have confirmed that air embolism in some patients may be associated with cardiopulmonary resuscitation (CPR) (17–21). However, air embolism resulting from this mechanism is exceedingly rare, with limited documentation in the literature, and its diagnosis requires stringent criteria: the patient must have an established underlying pathology responsible for the cardiac arrest, with the sequence of events being initial cardiac arrest due to the primary disease, followed by CPR, and subsequent identification of newly developed air embolism. Importantly, in such cases, air embolism is often not the direct cause of death but rather a byproduct of CPR—even in the absence of air embolism, the underlying pathology itself would be sufficient to cause fatality. The proposed mechanism involves positive pressure generated by artificial ventilation and chest compressions, forcing air through ruptured small pulmonary arteries into the circulatory system (21). Although existing evidence suggests that CPR may serve as a source of air embolism in extremely rare circumstances, this does not diminish its central role in the management of gas embolism. For the vast majority of patients, timely and effective CPR remains the critical intervention for life preservation and prognosis improvement. Chest compressions facilitate the expulsion of gas from the pulmonary arteries, maintain organ perfusion, and create a therapeutic window for hyperbaric oxygen therapy, ultimately enabling favorable outcomes (22). In our patient, the sudden cardiac arrest occurred without an identifiable underlying pathological basis, suggesting that cardiac arrest resulted from air embolism, with CPR being instituted subsequently. Based on this temporal sequence, we do not consider CPR to be the source of air embolism in our patient.

Unfortunately, the overt manifestations of air embolisms, including sudden onset of cardiovascular, respiratory, and neurological disturbances, are relatively nonspecific. When clinicians encounter patients who present with abrupt cardiopulmonary dysfunction, they often initially consider more common etiologies, such as pulmonary embolisms, cardiovascular events, or adverse reactions to anesthesia, which can lead to a delayed diagnosis of an air embolism.

Currently, multiple intraoperative monitoring modalities for air embolism are available in clinical practice. End-tidal carbon dioxide (ETCO₂) serves as a sensitive, non-invasive, and practical early monitoring indicator, with sudden decreases in ETCO₂ representing the first detectable clinical manifestation in some reported cases (23). However, the specificity of ETCO₂ is limited, as similar changes may occur in bronchospasm, airway obstruction, and low cardiac output states (24). Precordial Doppler ultrasonography demonstrates exceptionally high sensitivity for detecting gas emboli, capable of identifying minute bubble signals (25). Nevertheless, this technique is limited by a relatively high false-positive rate—the incidence of detected air embolism significantly exceeds that of clinically symptomatic cases, with the majority representing microemboli lacking hemodynamic significance (26). Consequently, the necessity for routine application of this monitoring modality during ERCP remains limited. Echocardiography also exhibits high sensitivity in air embolism diagnosis; bedside examination performed early in the embolic event enables direct visualization of intracardiac gas signatures, facilitating rapid definitive diagnosis and providing critical evidence for timely initiation of resuscitative measures (27). However, for the above monitoring modalities, ideal implementation is frequently precluded in clinical practice by factors including absence of essential equipment, prioritization of resuscitation efforts, and procedural delays.

This index case illustrates a scenario compounded by absent ETCO₂ monitoring and delayed echocardiographic examination. The limitation in intraoperative management was the failure to implement ETCO₂ monitoring, thereby missing the opportunity for early recognition of air embolism. Echocardiography was performed 95 min after symptom onset, by which time intracardiac gas had largely been absorbed or redistributed—precluding capture of gas shadows and yielding a false-negative result despite the high theoretical sensitivity of this modality. Postoperative echocardiography, however, demonstrated new-onset global hypokinesis of the ventricular wall, highly suggestive of myocardial ischemia secondary to coronary gas embolism, serving as indirect corroborative evidence of air embolism occurrence. This underscores the strict temporal constraints of ultrasonographic diagnosis, necessitating immediate execution upon suspicion of embolism. Definitive diagnosis was ultimately established by postoperative computed tomography, which detected intravascular gas bubbles. This lesson indicates that ETCO₂ should constitute standard configuration in high-risk endoscopic procedures to enable early warning, and any intraoperative ETCO₂ abnormality suspicious for air embolism should prompt immediate adjunctive confirmation via transthoracic echocardiography or precordial Doppler ultrasonography.

The present patient exhibited paradoxical blood pressure elevation during air embolism occurrence—a phenomenon rarely reported but documented in the literature. As early as the 1980s, this phenomenon was observed in animal experiments, with investigators attributing it to sympathetic nervous system activation and massive catecholamine release during early air embolism, resulting in transient severe hypertension (28). The neurosurgical literature similarly documents stress-related blood pressure elevation frequently accompanying early acute cerebral ischemia (29). It is hypothesized that the paradoxical hypertension in this case may be attributable to air embolism-induced acute cerebral ischemia triggering a sympathetic storm. Emphasis must be placed on the extreme rarity of this phenomenon in clinical reports; the classic hemodynamic manifestation of air embolism remains acute hypotension, and this case likely represents a special circumstance in which the early sympathetic compensatory phase was captured.

Hyperbaric oxygen therapy (HBOT) is widely recognized as an effective intervention for air embolisms, exerting therapeutic effects primarily through mechanisms such as reducing bubble volume (30).

Considering the severe consequences of air embolisms, prioritizing preventive measures is imperative. Studies have demonstrated that substituting carbon dioxide for air significantly reduces the incidence of complications (31). Moreover, inappropriate manipulation by surgeons during endoscopic insufflation of the gastrointestinal tract can result in excessive local pressure within the intestines, which is a recognized cause of air embolisms. Adjusting the insufflation pressure at the tip of the endoscope can further reduce the risk of gas embolisms (32).

## Conclusion

In summary, air embolism represents a potentially severe complication associated with ERCP. Its occurrence can result in significant physiological dysfunction and may pose a serious threat to patient safety. Therefore, during ERCP procedures, health care professionals should exercise caution to avoid the use of excessively high pressures and opt for carbon dioxide insufflation for gastrointestinal distension. For patients at high risk of air embolism, ETCO₂ monitoring should be implemented as a standard protocol to enable early detection, and any abnormal ETCO₂ findings warrant immediate confirmation through imaging modalities such as ultrasonography.

## Data Availability

The original contributions presented in the study are included in the article/supplementary material, further inquiries can be directed to the corresponding authors.
